# Editorial: The role of thyroid hormones in vertebrate development, volume II

**DOI:** 10.3389/fendo.2024.1408070

**Published:** 2024-04-17

**Authors:** Marco António Campinho, Laurent M. Sachs

**Affiliations:** ^1^ Algarve Biomedical Center-Research Institute, University of the Algarve, Faro, Portugal; ^2^ Faculty of Medicine and Biomedical Sciences, University of the Algarve, Faro, Portugal; ^3^ Unité Mixte de Recherche 7221, Département Adaptation du Vivant, Centre National de la Recherche Scientifique, Muséum National d’Histoire Naturelle, Alliance Sorbonne Universités, Paris, France

**Keywords:** thyroid hormones, development, vertebrates, teleosts, Amphibians, mammals

The thyroid hormone is an important signaling molecule system involved in vertebrate development, acting at the embryonic and post-embryonic levels (1). Although this has long been established, a detailed understanding of the thyroid hormone’s developmental action still needs to be fully understood. The present Frontiers in Endocrinology Research Topic aims to close this knowledge gap by bringing together a collection of papers addressing what is known and the challenges ahead on the role of thyroid hormones in vertebrate development, from teleosts to humans. The published manuscripts highlight the pivotal action of thyroid hormones in vertebrate neurodevelopment (Lee at al.; Ng et al.; O’Shaughnessy et al.; Richard et al.; Silva and Campinho; Valcárcel-Hernández et al.), post-natal development in amphibians (8–10) and how thyroid disorders can affect human development (Ru et al.; SeyedAlinaghi et al.).

Thyroid hormone signaling plays an essential role in neurodevelopment. Puzzling out the mechanisms of action of thyroid hormones in the developing brain will help contribute to progress in the prevention and treatment of several neurological disorders. Using data obtained in animal models, Richard et al. first review the adverse effects of thyroid hormone signaling disruption in the central nervous system before recalling how GABAergic neurons were found to be a major target of TH signaling during development and highlighting the difficulties in analyzing the mechanisms by which TH act on brain development. In mice, O’Shaughnessy et al. bring new data. They show how congenital hypothyroidism affects neurodevelopment at the ventricular zone, limiting glial cells, tight junctions, extracellular matrix, and blood-brain and blood-cerebrospinal barrier development. Moreover, this work suggests that the action of thyroid hormones in mice brain development likely occurs by genomic and non-genomic mechanisms. The action of thyroid hormones is not limited to the brain. The retina is also a target of thyroid hormones affecting cones, the photoreceptors that mediate color vision. Ng et al. previously reported the functions for thyroid hormone receptor TRb2 in mice, encoded by the gene *Thrb*. Here, they provide new knowledge investigating TRb1, another thyroid hormone receptor b isoform also expressed in the retina, suggesting novel functions in retinal and non-neural ocular tissues. Zebrafish is another animal model relevant to address the role of thyroid hormones in neurodevelopment. In this teleost fish, Silva and Campinho study the effect of functional impairment of T_3_ membrane transporter Mct8 (monocarboxylic acid transporter 8) that leads to the Allan-Herndon-Dudley syndrome when mutated in humans (2). The findings show that *mct8* gene deletion in zebrafish phenocopies many symptoms observed in patients, thus contributing to the understanding of the cellular and molecular mechanism underlying the severe underdevelopment of the central nervous system. The effect of thyroid hormones is also necessary for neurodevelopment throughout life. Valcárcel-Hernández et al. discuss the latest advances in the role of thyroid hormones in regulating adult neurogenesis in rodents and their potential implication in human health and therapeutical approaches in neurodegenerative diseases.

Another action controlled by thyroid hormones in vertebrates is the developmental change coming from a need to adapt to a change in habitat during post-embryonic development events. Amphibian metamorphosis is such transition from the larval state to the adult state, when the tadpole undergoes physiological and anatomical changes. This biological process resembles mammalian postembryonic development (3), with many organs mature into their adult forms. Using Xenopus, an anuran amphibian, Tanizaki et al. review the roles of thyroid hormones in larval epithelial cell death and *de novo* formation of adult intestinal stem cells, suggesting that T_3_-induced activation of cell cycle program. Still, in the Xenopus model, the work by Tribondeau et al. shows that T_4_ and T_3_ have similar actions and are involved in regulating the expression of genes involved in cell proliferation during early larval stages. Because these developmental stages are not normally subject to a physiological action of thyroid hormones, these data highlight the potential molecular actors that could be involved in the adverse effects of thyroid hormones during the larval period. The modalities of metamorphosis can be very diverse in amphibians, the latter also being paedomorphic by maintaining larval characteristics in reproductive adults and rarely, if ever, metamorphose in nature. This is the case of the Axolotl, the model used by Lazcano et al. Further expanding our knowledge on how different thyroid hormone metabolites are involved in post-natal development, they demonstrate bioactivity of 3,5-T_2_ inducing gill retraction, which is reversible following treatment withdrawal and at high concentration irreversible metamorphosis. These contrasting effects are explained by 3,5-T_2_ regulation of different genetic responses.

Finally, in human, Lee at al. investigate the iodine status and its association with thyroid function in South Korean children. Adequate iodine intake is essential because this trace element, is a necessary and limiting substrate for thyroid hormone synthesis and both deficient and excessive iodine status can result in thyroid dysfunction. They show that excess iodine was prevalent in Korean children and associated with a decrease in FT_4_ or T_3_ levels and an increase in TSH levels. In a different context, Ru et al. explore the association of maternal anti-thyroid peroxidase antibody (TPOAb) on placenta morphology and potential pathophysiologic inflammatory and oxidative stress responses in this organ and discuss the potential consequences for the developing embryo. Meta-analysis of previously published datasets by SeyedAlinaghi et al. reveals a close association between thyroid hormone disorders and later incidence of hip fractures in both men and women, shedding light on the effects of thyroid action through an individual lifespan.

With this Research Topic, we provide a diversified developmental and evolutionary picture of how thyroid hormones are important in vertebrate development. There is no doubt that further studies are required to fully understand the role of thyroid hormones during development not only in humans but also in many classes of vertebrates in order to better characterize and treat thyroid hormone-associated pathologies or anthropomorphic disruption of thyroid hormone signaling in humans (4) and ecosystems (5).

## Author contributions

MC: Writing – original draft, Writing – review & editing. LS: Writing – original draft, Writing – review & editing.
